# Design of a Novel Membrane Draft Tube Jet Loop Reactor (MDJLR) and Treatment of Slaughterhouse Wastewater

**DOI:** 10.3390/membranes9110155

**Published:** 2019-11-19

**Authors:** Burhanettin Farizoğlu, Süleyman Uzuner

**Affiliations:** Environmental Engineering Department, Engineering Faculty, Balıkesir University, 10145 Balikesir, Turkey; suzuner@gmail.com

**Keywords:** jet loop reactor, high compact reactor, ultra-filtration, membrane bioreactor, membrane fouling

## Abstract

The most important obstacle to the widespread use of membrane bioreactors (MBRs) is membrane fouling. In this study, a high-efficiency compact MBR was developed. Therefore, the draft tube of the jet loop reactor (JLB) was planned for use as a membrane module. The high-velocity jet streams, which are present according to the nature of the JLBs, provide high crossflow (cut-off force) on the membrane surface. Thus, the produced membrane module is operated in submerged membrane mode. This enhanced JLB modification is named the membrane draft tube jet loop reactor (MDJLR). This new system has a K_L_a value of 139 h^−1^ (at E/V of 2.24 kW m^−3^). In the next stage, treatment of slaughterhouse wastewater with the MDJLR was carried out. Under the 5.5 kg COD m^−3^ d^−1^ loading rate, efficiencies over 97% were achieved. The system operated continuously for 50 days without membrane backwashing or cleaning. During this period, fluxes of 3 L m^−2^·h^−1^ were approximately obtained at operating conditions of 850 mg L^−1^ MLSS (mixed liquor suspended solids) concentration, 1 bar suction pressure (∆P), and 3000 L h^−1^ circulation rate. This developed MDJLR will make jet loop membrane bioreactors (JLMBRs) and MBRs more compact and improve their performance.

## 1. Introduction

A jet loop bioreactor (JLB) is a concentric tubular reactor with two-phase coaxial nozzles located in the upper part of the reactor with an air inlet through the internal nozzle and a fluid inlet through the annular nozzle.

Due to the high efficiency of oxygen transfer under turbulent mixing conditions, the JLB has great potential, particularly for high organic strength biological wastewater treatment [1,2,3,4,5,6]. In addition, processing requires lower reactor volumes and a reduction in the required land area. Oxygen (air) is forcibly fed directly into the biological medium, resulting in significant savings in installation and maintenance costs [1]. Aeration of the liquid phase through the nozzles is carried out either in the ejector or injector modes. In the case of an ejector nozzle, the efficiency with respect to aeration is mainly determined by the absorption of gas into the flow of the driven fluid. Naturally, the air is sucked from the environment under the influence of a vacuum in the nozzle. This major drawback can be overcome by applying compressed gas (injector mode). However, additional power for aeration should be considered, especially in energy efficiency studies [7]. The use of jet loop reactors in different technologies and processes is being studied by many researchers due to the high mass transfer and mixing capabilities of these reactors [8].

To control a bioreactor at high biomass concentrations, a specific reactor topology should be selected. Jet loop bioreactors, which are high-speed, compact systems for an aerobic biological process, can provide an ideal reactor topology for an economical solution to industrial wastewater treatment.

One of the most serious problems associated with JLB is poor settling behavior caused by high nozzle shear and high food to microorganism ratio (F/M) [1,2,3,9,10]. To overcome these disadvantages, the hybridization of membrane units with JLB was proposed [9]. When a membrane module is coupled with a jet loop bioreactor, the bioreactor system is referred to as a JLMBR (jet loop membrane bioreactor).

The biofilm on the membrane in the MBR has always been a source of concern, but only as a hydraulic barrier [11,12,13,14,15]. Biofilm on the membrane surface creates resistance to the permeate flow. The formation of a biofilm on the surface of a membrane is unavoidable because all the microbial flocs, colloidal particles, and soluble organics in the mixed liquor are forced to move toward the membrane surface during the operation of the MBR. This kind of fouling increases operating costs by requiring constant replacement and cleaning of the membranes, increased aeration rate, and low permeate fluxes.

To prevent fouling, an aeration apparatus is usually placed under the membrane to deliver air bubbles to sweep the membrane surface. Some researchers have studied the effect of aeration on membrane fouling and filtration of wastewater. These researchers found that aeration generates shear stress exerted on the surface of the membrane, and this aeration lowers the filtration resistance of the MBR. Additionally, aeration causes turbulence and mixing in the reactor and this turbulence increases the shear stress on the surface of the membrane, thereby increasing the permeate flux rate. In a study on increasing or recovering flux, electrochemical processes were combined with MBR systems [16]. In these studies, flux values were significantly increased by using different electrodes (Fe and Ti anodes).

**Figure 1:**
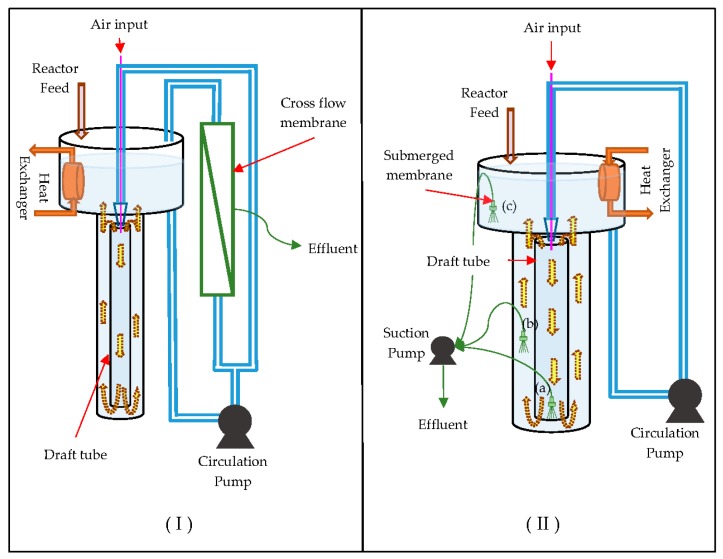
Schematics of (**I**) JLMBR (jet loop membrane bioreactor), side stream cross-flow membrane coupled jet loop bioreactor [17,18] and (**II**) MHCR (membrane-coupled high-performance compact reactor), submerged membrane modules placed in different regions of the jet loop bioreactor (e.g., (a) inside the draft tube, (b) outside the draft tube, and (c) inside the degassing tank) [19].

MBR configurations formed by membrane coupling to jet loop reactors can be divided into two configurations in the literature, as shown in Figure 1. The first of these configurations is JLMBR, which is a JLB coupled with a side stream cross-flow membrane [17,18], and the other is MHRC, which has submerged membrane modules placed in different regions of the high-performance compact reactor (HCR) [19]. In the JLMBR configuration, cross-flow created on the membrane causes shrinkage of flocks, so regular flock structures cannot form. This causes rapid membrane fouling [10]. In the MHCR configuration, submerged membrane modules were tested by placing them in the draft tube, between the draft tube and the reactor, and in the degassing tank. The highest permeate flux yields were obtained when the membrane module was placed in the draft tube [19].

In this study, the draft tube, which is one of the most important parts of the jet loop reactor, also functions as a membrane module. The jet stream containing air bubbles sweeps both the inner and outer surfaces of the draft tube, creating high shear forces. These shear forces not only ensure the removal of the biofilm on the surface, but also produce cross-flow to reduce the increased biofilm thickness. This developed reactor topology eliminates the problem of insufficient cross-flow, which is a significant disadvantage for submerged membranes. The jet flow produced here sweeps and cleans the entire surface of the membranes by passing tangentially. On the other hand, the JLMBR system will be more compact.

## 2. Materials and Methods 

### 2.1. Materials

#### 2.1.1. Membrane Draft Tube Jet Loop Reactor System Set-Up

**Figure 2:**
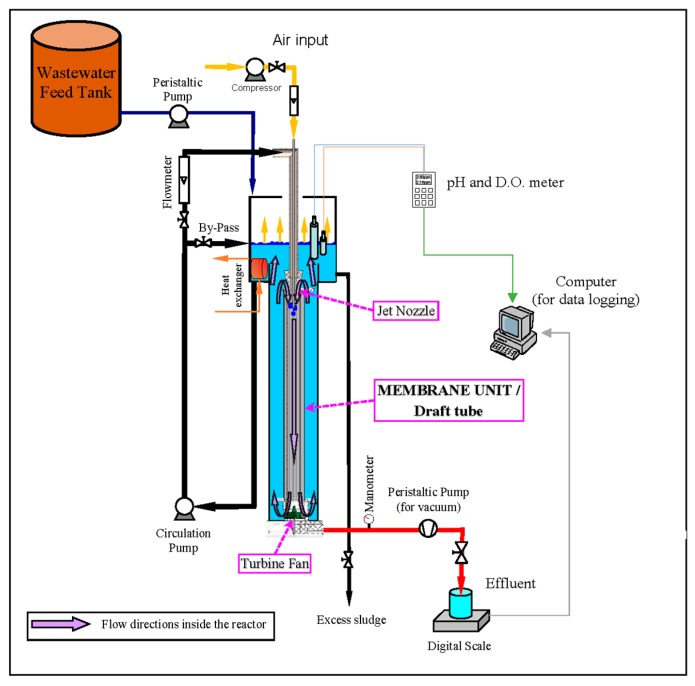
Schematic layout of the Membrane Draft Tube Jet Loop Reactor (MDJLR) reactor system.

The experimental system of the MDJLR (16 L capacity) was designed and developed at Balıkesir University, Environmental Engineering Department, Balikesir, Turkey. The jet loop reactor consisted of a cylindrical acrylic tube (height 1140 mm, the inner diameter 114 mm) with a height to diameter ratio of about 10:1 and included a draft tube membrane module that was open at both sides. The schematic impression of the reactor setup is given in Figure 2.

The nozzle, where air and water mix and are transferred into the reactor as a jet stream, was placed at the upper part of the reactor, causing a downward flow inside the draft tube membrane module. The nozzle was made from Teflon and included a centered stainless-steel tube (8 mm outer and 6 mm inner diameter). Air was supplied to the reactor via this stainless-steel tube from a compressor. A proportional valve and a rotameter were placed on the air supply line to adjust the air rate. Additionally, an electro-magnetic flowmeter and proportional valve controlled the liquid flow rate. The high-speed jet stream, which exits the jet nozzle and enters the draft tube, was pushed to the bottom of the reactor. This jet stream, which includes tiny air bubbles, impacts the turbine fan at the bottom of the reactor and then rises through the space between the draft tube and the reactor to the degasification tank. Additionally, at the top of the draft tube, the jet stream causes a low-pressure zone and then the air bubbles are sucked by this zone. This ongoing suction and jet stream creates a loop around the draft tube. This loop ensures a high oxygen transfer rate by keeping the air bubbles in the reactor for a prolonged time. The operating temperature of the reactor is kept fixed at around 20 ± 2 °C by using a stainless steel heat exchanger, which is immersed in the degasification section of the reactor.

#### 2.1.2. Draft Tube Membrane Filtration Module

The draft tube membrane module was constructed with 15 tube ceramic membranes (with one channel, 10 mm outer diameter, and 6 mm inner diameter). Atech Innovations Gbmh (Atech Innovations Gbmh, Gladbeck, Germany) supplied the membranes used in this study, which had pore sizes of 0.02 and 0.01 µm. Supplied membranes had a 1200 mm length and was cut to 390 mm lengths using a diamond coated saw blade in a band saw. The acrylic couplers and permeate collection apparatus were machined by a CNC milling machine using appropriate lengths of 10 × 10 cm acrylic stock. Additionally, custom silicone gaskets were molded to provide impermeability between the ceramic membranes and acrylic couplers. Permeate collection apparatus was connected to the bottom plate of the reactor via three stands, which were also used as permeate collection lines to the peristaltic pump. During the assembly of the draft tube membrane module, epoxy resin (two-part, 30-min, clear) and cyanoacrylate glue were used between the acrylic parts for leak proofing.

Table 1 shows the membrane (tubular, single channel, ceramic membrane) specifications. Effluent was measured with a digital scale (FZ-5000i, A&D Company Limited, Tokyo, Japan), which was placed on the permeate side. The flow readings were transmitted to a computer and recorded.

#### 2.1.3. Slaughterhouse Wastewater Characterization

The slaughterhouse wastewater (SWW) used in the experiments was obtained from the nearby Bigadiç Municipality Slaughterhouse. The characterization of slaughterhouse wastewater used in this study is shown in Table 2.

### 2.2. Methods

#### 2.2.1. Mass Transfer Analysis of the MDJLR

Power input, E, is calculated by converting the inlet water flow rates using this equation:

(1) $$E=Q_{L}\;\Delta P=Q_{L}\left(\rho_{L}\frac{u^{2}}{2}\right)$$

Here, Q_L_ is the input water flow rate in the jet nozzle; ΔP is the pressure drop converted to kinetic energy; ρ_L_ is the water density; and the water velocity exiting the jet nozzle is u. Then, the E/V values are calculated using Equation (1).

Tap water was used for all of the mass transfer tests. Each test was performed after dissolved oxygen (DO) in water was stripped down to below 0.2 mg L^−1^ by using nitrogen gas purge. Then, DO concentration was measured as a function of time using a multi-parameter (WTW Inolab 9430 IDS equipped with an FDO 925 DO probe). The FDO 925 probe also measured the temperature. The obtained data were sent to the computer for further analysis.

The overall volumetric mass transfer coefficient is K_L_a (the mass transfer coefficient) and can be used as a general expression of mass transfer. Using the following non-linear expression, K_L_a can be calculated:

(2) $$C={C_{s}}^{*}-\left({C_{s}}^{*}-C_{i}\right){\;e}^{-{(K}_{L}a)t}$$

where DO concentration at a given time (t) is C; C_s_* is the saturated oxygen concentration at the experimental conditions; and initial oxygen concentration is C_i_ (t = 0).

#### 2.2.2. Analysis of Membrane Fouling

A clean membrane’s permeation flux can be explicated by Darcy’s Law as:

(3) $$J=\frac{\Delta P}{{\mu \;R}_{m}}$$

In Equation (3), the permeate flux is J (m^3^ m^−2^ s^−1^); the absolute viscosity of the water is µ (Pa s); the clean membrane resistance (or hydraulic resistance of the clean membrane) is Rm (m^−1^); and ∆P (Pa) is the trans-membrane pressure (TMP). The permeation flux will always be lower than that calculated by Equation (3) for suspension filtration. Flux decline is a result of the increase in membrane resistance to the permeating flow, resulting from membrane fouling or particle deposition on or in the membrane [20].

#### 2.2.3. Analytical Methods

Inlet and outlet samples of the MDJLR system were taken daily. Parameters of MLSS (mixed liquor suspended solids), SS, TN, TP, COD, and BOD were analyzed according to standard methods [12]. The filtrate through Whatman GF/C glass-fiber filters was defined as dissolved COD, and was also used in the determination of MLSS. Temperature, pH, dissolved oxygen (DO), and conductivity probes were placed in the MDJLR and these parameters were measured via a multi-parameter (WTW Inolab 9430 IDS).

## 3. Results and Discussion

### 3.1. Design of the Draft Tube as a Membrane Module

The jet loop reactor design consists of two concentric cylindrical tubes. The jet nozzle, which is placed in the upper part of the reactor, is centered on the inner cylindrical tube. The inner cylindrical tube is named the “draft tube”. The draft tube and reactor must have an optimum diameter ratio of D_d_/D_r_ ≅ 0.4 for the maximum mass transfer rate. The jet nozzle is the apparatus where air and water are mixed and transferred into the reactor as a jet stream. The high-speed jet stream, which exits the jet nozzle, enters the draft tube, and is pushed through the draft tube to the bottom of the reactor. This jet stream, which includes tiny air bubbles, reaches the “impact plate” at the bottom of the reactor and then rises through the space between the draft tube and the reactor wall to the degasification tank. Additionally, at the top of the draft tube, the jet stream causes a low-pressure zone and then tiny air bubbles are sucked by this zone. This ongoing suction and jet stream creates a loop around the draft tube. This loop ensures a high oxygen transfer rate by keeping the air bubbles in the reactor for a prolonged time. In conventional reactors, the main purpose of the draft tube is to lengthen the water travel time so air bubbles stay in the water/reactor longer.

In this study, the intention was to provide a secondary function to the draft tube. The draft tube was designed for use as a membrane module.

**Figure 3:**
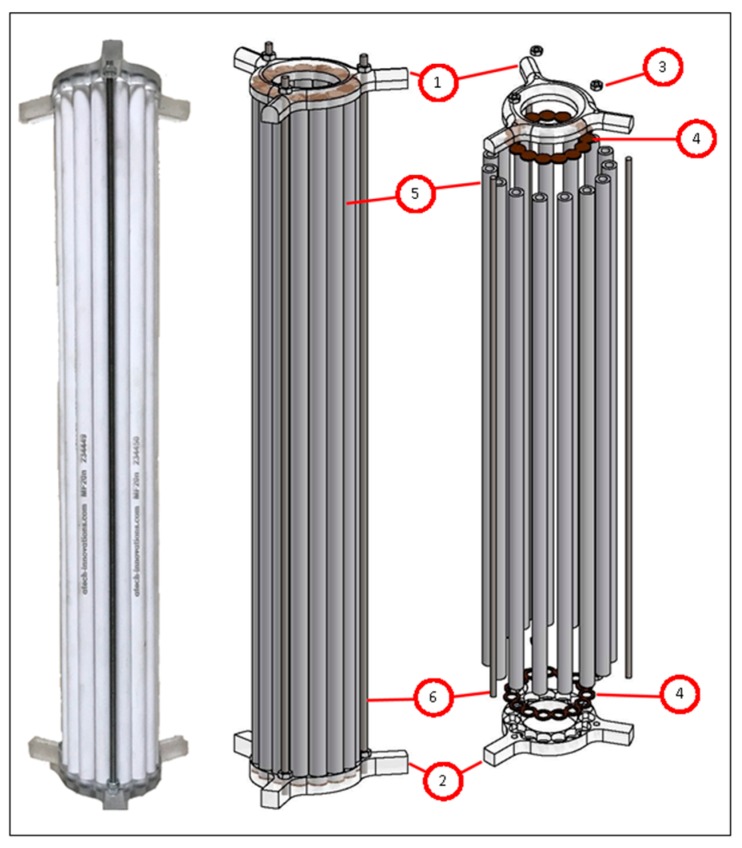
The ceramic membrane draft tube (1,2: CNC machined acrylic couplers; 3: M4 stainless-steel nut; 4: custom-molded silicone gaskets; 5: ceramic membranes; 6: stainless-steel M4 threaded rods).

In the first stage of this study, a draft tube with an optimum diameter ratio to the reactor (D_d_/D_r_ = 0.4) was engineered and produced by using single-channel ceramic membranes, which had a 10 mm outer and 6 mm inner diameter. The produced ceramic membrane module draft tube was 5 cm in diameter. The ceramic membrane draft tube is seen in Figure 3. 

**Figure 4:**
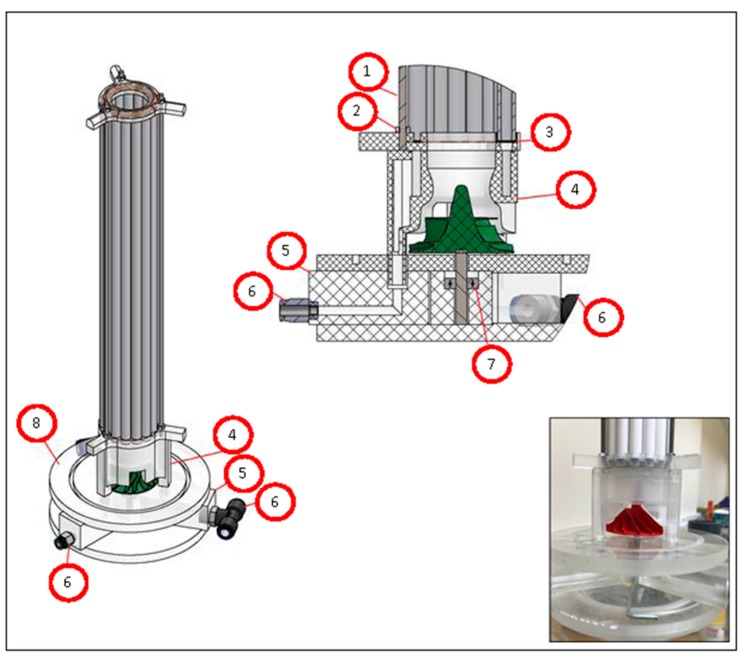
The permeate collection apparatus and the turbine fan as an impact plate (1,2: stainless-steel M4 threaded rod and nut; 3: custom-molded silicone gasket; 4: CNC milled acrylic permeate collection apparatus; 5: acrylic block with “L” shaped hole; 6: tubing connections; 7: stainless-steel rod and bearing; 8: reactor bottom plate).

In addition, some acrylic couplers were engineered and produced for the draft tube’s upper and bottom ends to maintain the circular geometry (Figure 4). These acrylic couplers also provide impermeability and allow the collection of the permeate. A peristaltic pump, which creates a vacuum, was connected to the bottom acrylic coupler of the draft tube membrane module to collect the permeate. By doing this, the created vacuum transfers water to the inside of the membrane and leaves the particulate matter in the reactor suspension.

This membrane process is operated as a submerged membrane process. The submerged membrane process operating procedure includes surface cake layer cleaning by creating a cross flow at the surface of the membrane. This cross-flow is generally created by placing air diffusers at the bottom of the membrane tank. The parallel movement of air bubbles along the membrane surface creates a cut force to remove the cake layer. On the other hand, another approach for submerged membrane process operation is rotary disc membranes. This modification relies on rotary action to sweep the cake layer off the surface of membranes. The goal of conventional approaches is to create cross-flow either by using air bubbles or rotary action. The biggest disadvantage of operating the submerged membrane process is the inability to create enough cross flow to sweep the surface of membrane, so the thickness of the cake layer increases and this causes low permeate fluxes.

**Figure 5:**
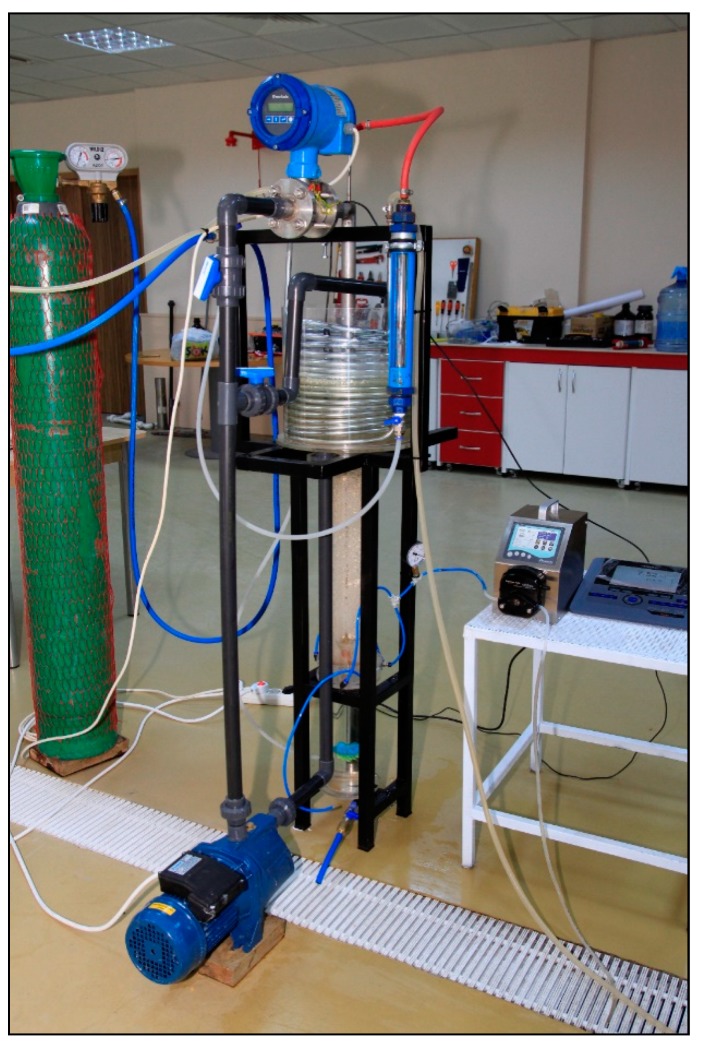
The developed membrane draft tube jet loop reactor.

In this study, according to the jet loop reactors’ nature, high-velocity jet streams create high cross-flow cut force inside and outside the produced draft tube membrane module. Air bubbles carried by the jet stream are forced through the inside of the draft tube membrane module to the bottom of the jet loop reactor and create high speed (avg. 1.75 m s^−1^) cross-flow cut force on the surface of the membranes. Then, this jet stream reaches the centered turbine fan and rotates the turbine fan at the bottom of the reactor, the rotational movement of the turbine fan homogenizes the jet stream and air bubbles. Then, the water rises outside the draft tube membrane module. While this rising occurs, air bubbles create cross-flow outside the draft tube membrane module. Thus, uniform cross-flow is produced. High-speed cross-flow sweeps both the inner and outer surfaces of the draft tube membrane module and lowers the accumulation rate of the cake layer. Figure 5 shows the developed MDJLR that was engineered and manufactured in this study.

The operating range of circulation changed between 2500 to 4500 L h^−1^ and air rates were 400 to 4500 L h^−1^. At these operating conditions, cross-flow velocities on the inner side of the draft tube/membrane module were between 0.71 to 2.20 m s^−1^ and at the outer side, they were between 0.11 to 0.35 m s^−1^. The cutting forces were applied to the membrane surface in direct proportion to these cross-flow velocities. Additionally, this is much higher than the cross-flow rate in classical submerged MBR systems. Hence, this will prevent and reduce the accumulation of the cake layer on the membrane surface.

This approach and design is an original and unique idea. Therefore, this designed MDJLR could eliminate the disadvantages of submerged membrane processes. However, JLMBR studies in the literature are about industrial wastewater treatment and these studies show that the membrane module is mandatory for jet loop reactor treatment performance, because flocks formed in jet loop reactors are very small and have weak or no settleability. It is known that jet streams are very effective at shrinking flock. Additionally, cross-flow created in the external membrane module causes flocks to be even smaller. This leads to a permeate flux rate decrease for the system. In this study, no cross-flow was additionally created. It is expected that this will lead to a better permeate flux rate.

### 3.2. The Mass Transfer Capacity of the MDJLR

**Figure 6:**
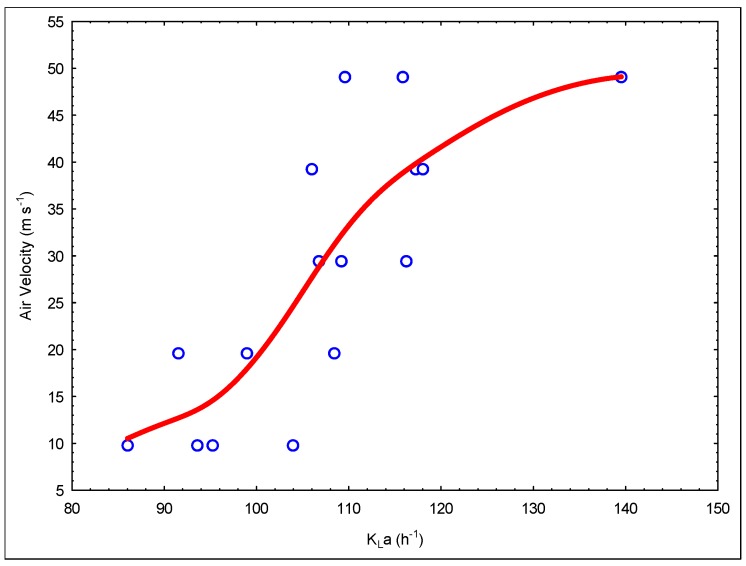
The change in the K_L_a via Q_air_/Q_liquid_ rate.

**Figure 7:**
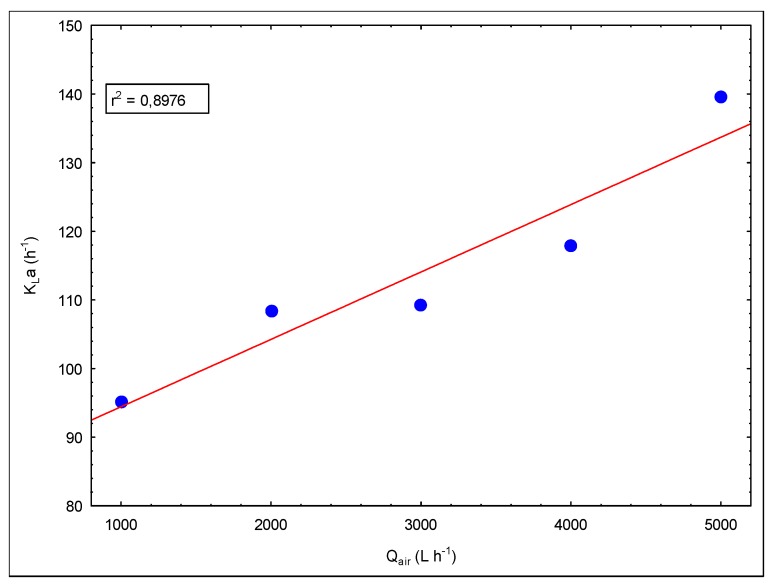
The evolution of K_L_a versus air flow rates (E/V = 2.2377 kW m^−3^).

The performance of biological systems is directly related to the chosen reactor type. Mixing of the reactor and oxygen transfer properties are very important in system performance, especially in aerobic processes. Jet loop reactors have good mixing properties and high oxygen transfer rates, and this provides a great advantage for high strength industrial wastewater treatment. At this stage of the study, the mass transfer properties of the engineered reactor modification were inspected. Figure 6 shows the change in K_L_a values via the Q_air_/Q_liquid_ rate and Figure 7 presents the change in K_L_a values to air flow rate.

The increase in the circulation rate enhances the energy transferred to the reactor. Thus, this increases cut forces, causing shrinkage of the air bubbles, which enhances the mass transfer rate. At the same time, the air rate rise increases the gas holdup, which causes an augmentation of the air/water cross-section area. This increment increases the K_L_a values. 

**Figure 8:**
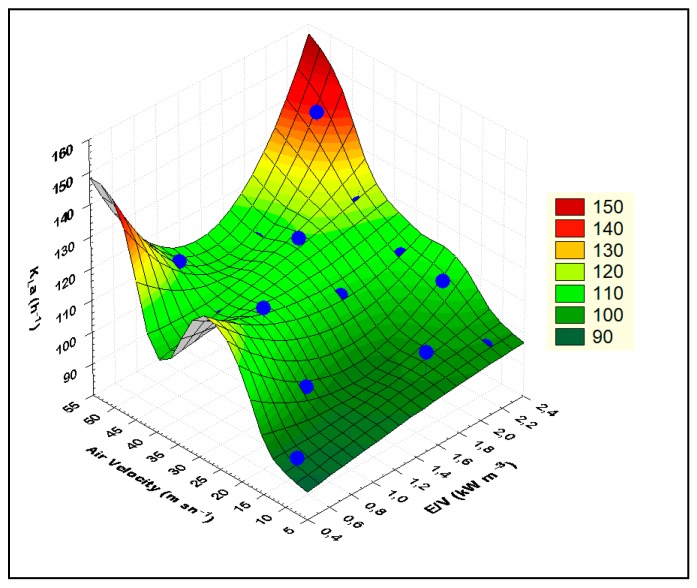
Mass transfer coefficient (K_L_a) model expression (Statistica 6.0).

**Figure 9:**
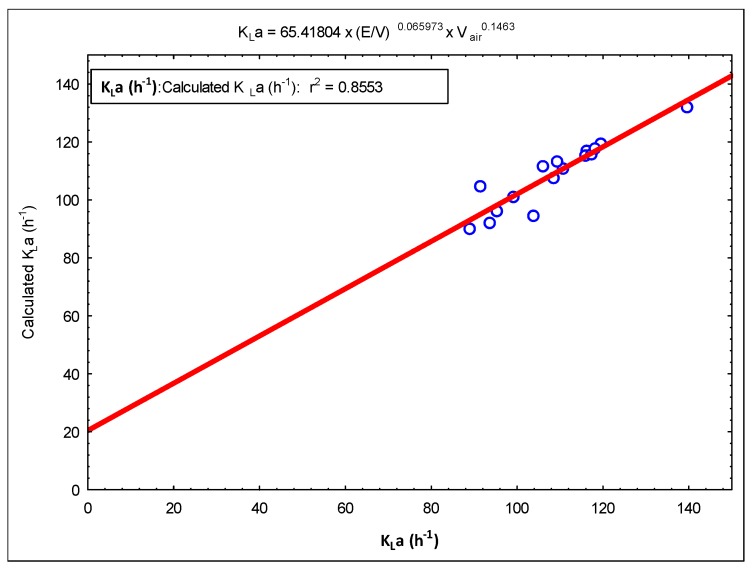
Calculated K_L_a versus measured *K_L_a* (Statistica 6.0).

A simple model was constructed using these K_L_a data. Figure 8 and Figure 9 show graphs of this model. The following model expression is given as a result of these modeling studies.

(4) $$K_{L}a=65.41804\times {\left(E/V\right)}^{0.065973}\times {V_{\mathrm{air}}}^{0.1463}$$

In the mass transfer studies, *K_L_a* values were measured between 86 and 139 h^−1^. These values are smaller than the values obtained in previous studies [10]. The reasons for this are interpreted as due to the difference in operating ranges and parameters. It was observed that the jet stream, which is driven through the draft tube/membrane module, carries tiny air bubbles, and these air bubbles leak through the openings between the ceramic membranes (nearly 1 mm) along the length of the draft tube/membrane module. This situation was also observed and detected during experiments using tap water in this acrylic reactor setup. Due to the leakage of air bubbles, air bubbles escape to the degasification tank in less time, which decreases the air retention time, causing a decrease in the mass transfer rate. In addition, the occurrence of biofilm between ceramic membranes was observed and this blocks the pathways of air bubble leaks. Therefore, this negative situation will not be observed during operation.

On the other hand, the engineered draft tube/membrane module had a thickness of 1.2 cm. This thickness decreases suction at the upper part of the draft tube/membrane module, where rising air bubbles are sucked into the draft tube/membrane module. This reduces the number of bubbles entering the loop and thereby reduces the air retention time in the system, and has a negative effect on mass transfer.

### 3.3. Treatment Performance of MDJLR and the Efficiency of the System

#### 3.3.1. Slaughterhouse Wastewater Treatment Performance of MDJLR

In this stage of the study, slaughterhouse wastewater was used in the developed reactor system for treatment. Experiments still continue for this reactor system. Increasing jet velocity in the MDJLR increases the energy consumption. Therefore, operating parameters for the system should be set to minimize jet velocities. The mixed liquor in the reactor could not be recirculated at lower jet velocities, and the loop did not form in the MDJLR. Therefore, a fixed air flow rate, the minimum jet velocity, was selected for the liquid (reactor mixed liquor) loop.

**Figure 10:**
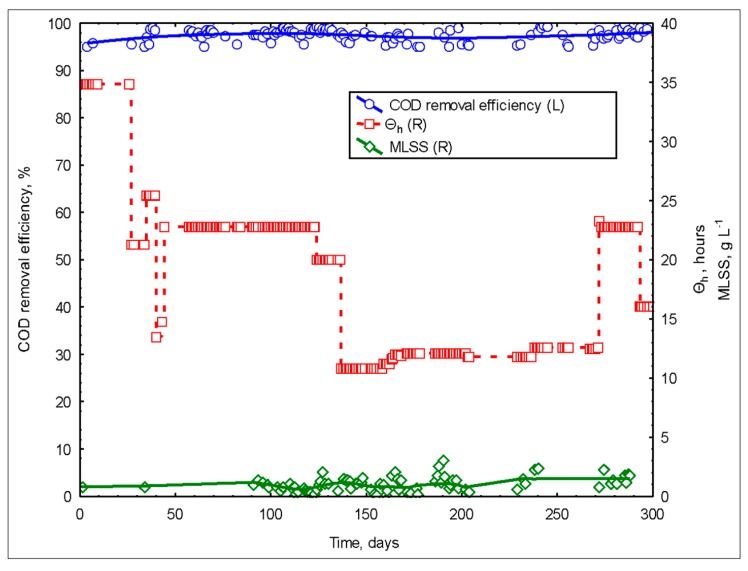
The change of COD removal efficiency over time via a change of MLSS (mixed liquor suspended solids) and HRT (hydraulic retention time).

The reactors’ circulation rate was adjusted to 3000 L h^−1^. The system air inlet was provided in the ejector mode because the ejector mode supplies enough air to keep dissolved oxygen concentrations over 3.5 mg L^−1^. The activated sludge in the system was taken from the aeration tank of a well-operated activated sludge system. The system was operated at batch mode until the MLSS concentrations reached 350 mg L^−1^, then continuous operation began. SWW fed directly into the system without any pretreatment. The obtained treatment efficiencies are shown in Figure 10. Very high treatment efficiencies were obtained from the system throughout the entire study. Effluent had a COD concentration under 100 mg L^−1^ with every operating condition of the reactor system.

High fluctuations in the inlet loading rates resulted in minimal reductions in the system performance. Overall, MDJLR showed a high tolerance for short term changes in high COD loading rates [21]. MDJLR had higher F/M values than the conventional activated sludge systems and higher active bacteria growth rate conditions, which can be beyond the limits at which filamentous organisms can compete successfully with the rest of the population [2].

While feeding with high influent COD concentrations and changing the reactor loading rates, foaming occurs. In other words, high F/M ratios cause excessive foaming in the bioreactor. This resulted in low MLSS concentrations in the system. The foaming decreased to a minimum level when the system reached steady-state conditions.

On the other hand, the occurrence of excessive biofilm formation was observed on the reactor walls, membrane surfaces (draft tube), and inside of the degasification tank. The occurred biofilm formation in the degasification tank was cleaned by hand and passed to the suspension 2–3 times a day. However, especially on the reactor walls and surface of the ceramic membranes/draft tube, rather thick biofilm formation was observed. This caused the system to operate as a hybrid system and enabled high performance even at low MLSS concentrations. Due to the strong aerobic and active bacterial populations in JLMBR systems, high performance was achieved even at low MLSS concentrations. However, the pore diameters of the selected membranes were very small (0.01–0.02 µm), allowing high yields even at low MLSS values. Both the low power of the selected vacuum pump and the formation of sludge in the very small flock structure caused low fluxes from the system. This had a negative effect on increasing loading rates. Thus, the hydraulic balance in the system was established by selecting low feed rates. This resulted in high hydraulic retention times (HRT).

During this study, the MDJLR system had total nitrogen (TN, NH_4_-N) and total phosphorus (TP) removal efficiencies of 68 and 63%, respectively.

#### 3.3.2. Ultrafiltration of the Sludge

**Figure 11:**
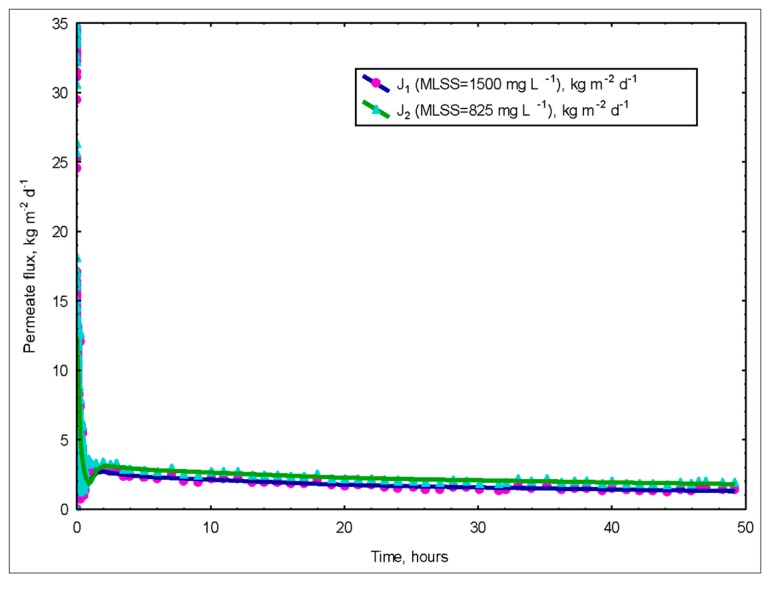
Variation of fluxes over time at various MLSS concentrations in the membrane unit (ΔP = 1 bar).

Settling tanks (clarifiers) lead to deterioration of the effluent when the activated sludge has poor sedimentation characteristics. Therefore, even if the biological transformation is very good, if the sludge does not settle well, a good treatment quality cannot be realized. By using membranes, the sludge settling problems were eliminated [20,21]. On the other hand, because of the high shear forces, JLBs cause sludge formation in very small and dispersed flock structure. This creates a high effective surface area in the interaction of oxygen and organic substances with the flocks, but in most cases, leads to the formation of flocks that have an undetectable sludge volume index (SVI). In this case, the real high performance of the system can be achieved by adding the high separation performance of the membranes to the reactors. Figure 11 shows the variation of fluxes with time according to MLSS concentrations.

TMP (transmembrane pressure) was kept constant during the study period. Approximately 50 d after the insertion of the membrane module, it was removed and cleaned, the membranes were inserted and the process continued as before. The membrane exchange process takes about 3 h. Meanwhile, the activated sludge was taken to another tank and aerated.

After the reactor setup, membrane studies continued. As can be seen, the permeate flux rates were quite low. The first reason for this is that the peristaltic pump used in the system created a maximum vacuum of negative 1 bar. The other reason is that a biofilm layer formed on the surface of the ceramic membranes due to excessive accumulation of bio matter.

In the next stage of the study, the draft tube membrane module was manufactured using flat membranes and the study is still ongoing. This change in membrane-type is an improvement to the system and grants an increase in membrane flux rates by doubling the flux rate.

Experiments on the membrane fouling mechanisms are ongoing.

## 4. Conclusions

The following findings were obtained in this study, which are new options for both MBR technology and high-speed new generation reactors.

1. JLBs which are high-speed compact reactors, are increasingly used for treating high-strength wastewaters. The inclusion of membrane technologies into the JLBs is very important for small and unstable flock removal and this inclusion enhances performance. There are previous studies in the literature using both submerged membranes, which are placed at different regions of the JLB (M-HCR) and external cross-flow membrane modules (JLMBR).
2. In this study, according to the nature of the jet loop reactor, high-velocity jet streams were used for creating high cross-flow cut force. For this reason, the draft tube of the reactor was produced as a membrane module and the intention was to gain a secondary function for the draft tube.
3. This developed modification was named as a membrane draft tube jet loop reactor (MDJLR).
4. High cross-flow is supplied to the membranes’ surface with high-speed liquid (water and air) flow by using the produced MDJLR. High-speed cross-flow sweeps both the inner and outer surfaces of the draft tube membrane module and prevents the accumulation of a cake layer.
5. Mass transfer properties of the MDJLR were investigated and quite high K_L_a values (86–139 h^−1^) were obtained (at E/V value of 2.24 kW m^−3^).
6. In this study, the treatment of slaughterhouse wastewater was investigated with the developed MDJLR. The system had a treatment efficiency of over 97% at a 5.5 kg COD m^−3^ d^−1^ loading rate.
7. The system operated continuously for 50 days without membrane back washing or cleaning. At the fiftieth day, a flux rate of 3 L m^−2^ h^−1^ observed under operating conditions of 850 mg L^−1^ MLSS concentration, 1 bar ΔP, and 3000 L h^−1^ circulation rate.
8. The most important parameter that restricts the studies was the inability to reach high ΔP values. Therefore, high flux values and high loading rates could not be reached.

A back-washing unit will be coupled to the system. When this is realized, it is predicted that the flux values can be kept around 15 L m^−2^ h^−1^ over a long period of operation.

The membrane properties of the produced MDJLR are still being studied. It was observed that the ceramic membranes used clogged quickly. In the second stage of the study, the draft tube membrane module was changed to a new design by using another type of membrane and is still being studied. The results obtained will be shared in future literature studies.

## Figures and Tables

**Table 1:** Specifications of the ceramic membranes used in the system.

Support Material		α-Al_2_O_3_			
Membrane material		MF: α-Al_2_O_3_; ZrO_2_; TiO_2_; UF: TiO_2_; ZrO_2_; Al_2_O_3_			
Pore diameter/Molecular weight cut off		0.02; 0.01 µm; 1 kD			
pH stability		0 to 14			
All membranes are suitable for steam sterilization ≥ 121 °C/249.8 °F					
Type	Design (mm)	Amount of channels	Length (mm)	Filter surface per element (m^2^)	Illustration
1/6	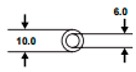	1	1200	approx. 0.023	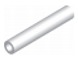

**Table 2:** Characterization of the slaughterhouse wastewater (SWW) used in this study.

Parameter	Concentration (mg L^−1^)			
	Min	Max	Avg.	Standard Dev.
Total COD	906	9939.4	2824.50	974.35
Dissolved COD	742.5	4577.75	2477.92	892.08
Dis. COD/T.COD	0.57	1.00	0.88	0.1007
BOD	325	8145	2173	627.12
Suspended Solids (SS)	40	520	155.80	96.086
Total Solids	44	13,635	2689.54	2317.72
Total Nitrogen (TN)	48	632	213.67	163.82
Total Phosphate (TP)	20.3	132.5	54.2	33.76
pH	5.9	7.8	6.3	1.24
